# Persistent Hyperschematia With Over-Generation Following Recovery From Unilateral Spatial Neglect: A Case Report

**DOI:** 10.7759/cureus.77951

**Published:** 2025-01-25

**Authors:** Risa Yoshikawa, Yuji Minamikawa, Michihiro Osumi, Shu Morioka

**Affiliations:** 1 Physical Medicine and Rehabilitation, Graduate School of Health Sciences, Kio University, Nara, JPN; 2 Physical Medicine and Rehabilitation, Nishiyamato Rehabilitation Hospital, Kanmaki-Cho, JPN; 3 Pain Medicine, Graduate School of Health Sciences, Kio University, Nara, JPN

**Keywords:** hyperschematia, inferior fronto-occipital fasciculus, middle longitudinal fasciculus, over-generation, spatial cognition, stroke, unilateral spatial neglect

## Abstract

Hyperschematia is characterized by excessive representation of left-sided space and typically improves alongside unilateral spatial neglect (USN). The role of white matter disconnection, particularly involving ventral visual pathways, in the persistence of hyperschematia remains poorly understood. We report a case of a 44-year-old right-handed man who developed USN following a right putaminal hemorrhage. While his USN symptoms improved with rehabilitation, he exhibited persistent hyperschematia characterized by a unique "over-generation" phenomenon, where he produced excessive elements in drawing tasks (e.g., additional flower petals and clock numbers). Notably, this occurred without accompanying asomatognosia. Neuroimaging and disconnection analysis revealed significant disruption of the inferior fronto-occipital fasciculus (IFOF) and middle longitudinal fasciculus (MdLF), suggesting impairment of ventral visual processing pathways. The Behavioural Assessment of the Dysexecutive Syndrome (BADS) indicated executive dysfunction, particularly in tasks requiring feature monitoring and boundary maintenance. While the patient showed improvement in spatial awareness on the Catherine Bergego Scale, the "over-generation" phenomenon persisted throughout the rehabilitation period. This case suggests that hyperschematia can persist independently of USN recovery and without body schema disturbances. Ventral pathway disruption may impair integration between visual object processing and frontal monitoring systems, contributing to persistent over-generation. These findings highlight potential neural mechanisms underlying hyperschematia and inform targeted rehabilitation strategies.

## Introduction

Unilateral spatial neglect (USN) is a well-recognized disorder following right hemisphere damage [1,2]. Traditionally, USN is described as a “negative symptom”, primarily reflecting a failure to attend or respond to contralesional (usually left) hemispace [1,3]. For clarity, "contralesional hemispace" refers to the side of space opposite the side of the brain lesion; in the case of right hemisphere damage, this would typically be the left side of the patient's visual or extrapersonal space. However, recent studies suggest that USN may also include "positive symptoms" such as allochiria and hyperschematia [4,5]. Allochiria is characterized by the transposition of perceived stimuli from one side to the other (e.g., perceiving a left-sided touch as occurring on the right), whereas hyperschematia is an exaggerated representation of space or body parts.

Hyperschematia is particularly intriguing as it manifests as an over-expansion of contralesional space and body representation [6-8]. Rode characterized this phenomenon as a distortion of the internal spatial framework rather than a simple attentional bias [9]. Although hyperschematia typically improves in parallel with USN and often co-occurs with asomatognosia or anosognosia, cases in which hyperschematia persists after resolution of neglect are remarkably rare [6-8,10]. Asomatognosia refers to the loss of recognition or awareness of one's own body parts, whereas anosognosia refers to the lack of awareness of one's own illness or deficits. These related but distinct conditions may accompany USN, further complicating the clinical picture.

Recent neuroimaging studies have underscored the pivotal role of white matter disconnection in spatial cognition [11-13]. While early work focused on the superior longitudinal fasciculus (SLF) and dorsal stream mechanisms, emerging evidence emphasizes the importance of ventral pathways in mediating higher-order visual and executive processes [14,15]. These include the inferior fronto-occipital fasciculus (IFOF) and the middle longitudinal fasciculus (MdLF), which link occipital/temporal regions to frontal and parietal areas [11,12,16-18]. Disruptions in these pathways may lead to complex phenomena that transcend classical neglect, potentially affecting body representation and feature processing. Interestingly, many reports of hyperschematia have described concurrent body schema disturbances, such as asomatognosia or disownership of limbs, highlighting the intertwined nature of spatial representation and bodily awareness [19,20].

Here, we describe a unique case of persistent hyperschematia exhibiting a distinctive “over-generation” phenomenon even after the patient’s USN had improved. Unusually, no clear asomatognosia was observed, challenging the assumption that hyperschematia necessarily coexists with body schema disorders [6-8,19,20]. By integrating detailed cognitive assessments with white matter disconnection analysis, we aim to illustrate how ventral pathway involvement, particularly in the IFOF and MdLF, may underlie this rare presentation, offering important implications for rehabilitation strategies.

## Case presentation

Basic information

A 44-year-old right-handed male (height: 170 cm, weight: 79.3 kg, BMI: 27.4) was admitted to our emergency department after experiencing leftward deviation while walking and subsequent collapse. He worked as a sales representative, which required frequent driving and client visits. His medical history included untreated hypertension for approximately one year and right shoulder dislocation one month prior, which had resolved without complications. He had no family history of stroke. He was an occasional drinker and a current smoker (20 cigarettes/day). The patient lived alone and unmarried, and his primary concerns were "I want to return to work as soon as possible" and "I want to regain movement in my left arm and leg." Written informed consent was obtained from both the patient and his family for the publication of this case report and accompanying images, with all personally identifiable information minimized for privacy protection.

Clinical findings

Initial neurological examination revealed consciousness disturbance (Japan Coma Scale: JCS 10), right conjugate deviation, left hemiplegia (Brunnstrom Recovery Stage I for upper extremity (UE), lower extremity (LE), and hand), left hemisensory loss, and dysarthria. The National Institutes of Health Stroke Scale (NIHSS) score was 19. Laboratory tests revealed elevated blood glucose (248 mg/dL) and HbA1c (7.3%), leading to a new diagnosis of diabetes mellitus, for which metformin therapy was initiated.

Imaging findings

**Figure 1:**
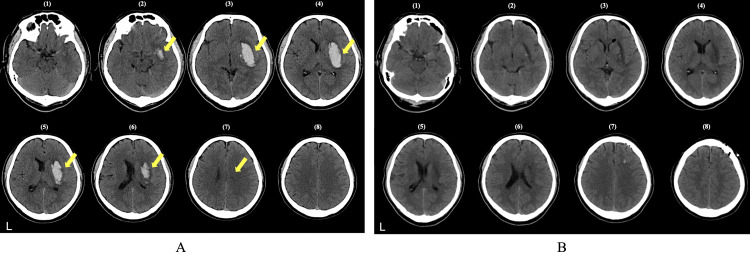
Preoperative and postoperative axial CT Images (A) Preoperative CT scans: Axial CT slices labeled (1) through (8), obtained upon admission, demonstrate a right putaminal hemorrhage (yellow arrows) measuring approximately 50 mL. The hematoma extends into the putamen, external and internal capsules, and the insular cortex. Each numbered slice corresponds to a successively higher axial level, highlighting the cranio-caudal extent of the hemorrhage. (B) Postoperative CT scans: Axial CT slices labeled (1) through (8), obtained 24 hours after emergency hematoma evacuation, show that the previously observed hyperdense hemorrhage is no longer visible (compare each slice with its corresponding slice in (A)), confirming successful evacuation of the hematoma. Mild postoperative changes, including subtle ventricular shifts, may be noted; however, there is no evidence of rebleeding or significant residual hematoma. Note: The slice numbers (1) through (8) in both (A) and (B) are matched by approximate axial levels to facilitate direct comparison of preoperative and postoperative scans. These CT images have been left-right reversed by MRIcron to maintain a consistent orientation with subsequent figures.

**Figure 2:**
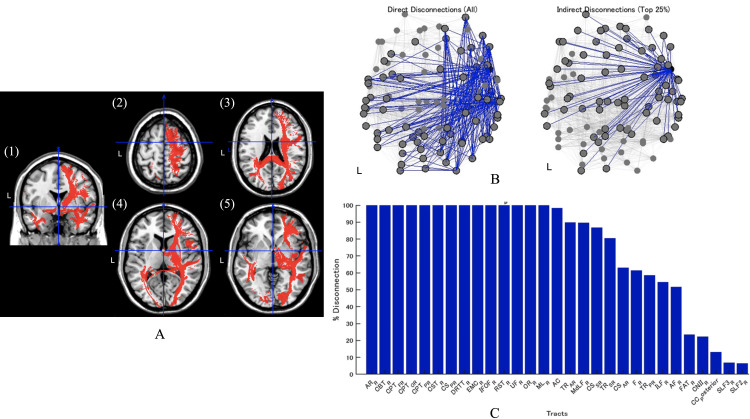
White matter disconnection analysis (A) Images illustrating the lesion distribution (in red). Panel (1) shows a coronal slice, while panels (2)-(5) depict axial slices. Crosshairs indicate the analysis coordinates. Estimation of damaged fibers in these slices indicates severe disruption (in some tracts >90%) in the AR, CBT, CPT, IFOF, MdLF, and others. (B) Graphical representations of the structural network disruptions from the connectome analysis. The left panel (direct disconnections) shows direct disruptions to neuronal pathways (blue lines indicate disrupted connections), whereas the right panel (indirect disconnections) highlights the top 25% of indirect disruptions stemming from these direct lesions. (C) A bar graph displaying the percentage of disconnection for each white matter tract. The horizontal axis lists the tract names, and the vertical axis indicates the percentage of disruption. Higher bars denote a greater proportion of disruption within that tract. AR, acoustic radiation; CBT, corticobulbar tract; CPT, corticopontine tract; CST, corticospinal tract; CS, corticostriatal tract; DRTT, dentatorubrothalamic tract; EMC, extreme capsule; IFOF, inferior fronto-occipital fasciculus; RST, reticulospinal tract; UF, uncinate fasciculus; OR, optic radiation; ML, medial lemniscus; AC, anterior commissure; TR, thalamic radiation; MdLF, middle longitudinal fasciculus; F, fornix; ILF, inferior longitudinal fasciculus; AF, thalamic radiation; FAT, frontal aslant; CNII, cranial nerve II; CC, corpus callosum; SLF, superior longitudinal fasciculus; Subscript R, right; Subscript F, frontal; Subscript O, occipital; Subscript P, parietal; Subscript A, anterior; Subscript P, posterior; Subscript S, superior

An initial computed tomography (CT) scan revealed a right putaminal hemorrhage (hematoma volume: 50 mL) involving the putamen, as well as the external/internal capsules and the insular cortex (Figure 1A). Emergency hematoma evacuation was performed approximately five hours after onset, and a post-operative CT scan at 15 hours confirmed successful removal of the hematoma (Figure 1B). After the brain lesion area was delineated using MRIcroN (McCausland Center for Brain Imaging, Columbia, US), the images were standardized using SPM12 in MATLAB (MathWorks, Massachusetts, US). Neural network analysis was then conducted using the Lesion Quantification Toolkit [13]. This method estimated the affected white matter fibers and mapped pathways with disconnections. The analysis revealed extensive white matter disconnection, with particularly severe (>90%) disruptions in tracts such as the acoustic radiation, corticobulbar tract, corticopontine tract, IFOF, and MdLF (Figure 2).

Rehabilitation intervention and clinical course

**Figure 3:**
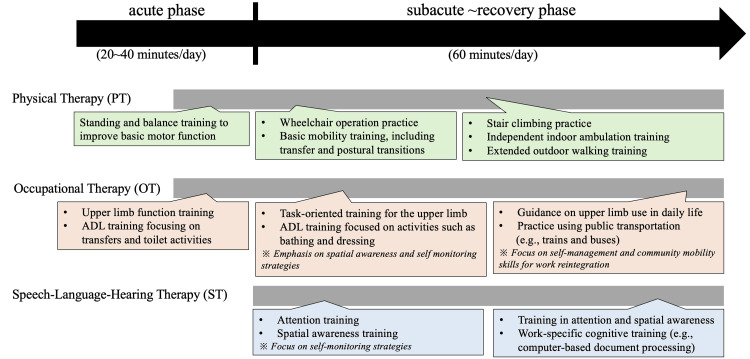
Comprehensive rehabilitation timeline and interventions Timeline showing the progression of rehabilitation interventions from acute to recovery phase. PT progressed from basic motor function training to independent ambulation. OT focused on upper limb function and activities of ADL, with emphasis on spatial awareness and self-monitoring strategies in preparation for work reintegration. ST addressed attention, spatial awareness, and work-specific cognitive skills. Green boxes indicate PT interventions progressing from basic mobility to advanced functional training. Pink boxes show OT interventions advancing from basic ADL to community mobility. Blue boxes represent ST interventions focusing on cognitive and work-related skills. Therapy duration increased from 20-40 minutes per day in the acute phase to 60 minutes per day in the subacute to recovery phase. Italicized notes highlight specific therapeutic strategies focusing on spatial awareness, self-monitoring, and work reintegration goals. PT, physical therapy; OT, occupational therapy; ADL, daily living; ST, speech-language-hearing therapy

The timeline of key clinical events and interventions is shown in Figure 3. Rehabilitation was initiated on postoperative day two following confirmation of clinical stability. On day 24, the patient was transferred to our rehabilitation unit. Physical therapy (60 minutes daily) initially focused on improving basic movement abilities, progressing from sit-to-stand and standing balance exercises to indoor walking training, and eventually to outdoor walking and stair climbing. Occupational therapy (60 minutes daily) initially emphasized activities of daily living (ADL) independence through task-oriented training of the paretic upper limb and practice of toileting and dressing activities. Prior to discharge, occupational therapy focused on return-to-work training (commuting practice), with consistent attention to spatial awareness and self-monitoring strategies throughout the intervention. Speech therapy (60 minutes daily) initially focused on cognitive training, particularly spatial awareness and attention functions, progressing to work-related training (desk work) during the middle phase of hospitalization. At admission to our rehabilitation unit, the patient demonstrated severe hemiparesis with Fugl-Meyer Assessment (FMA) scores of 5 for the UE and 3 for the LE. The patient was discharged on day 201. Although the FMA-UE score improved to 14 at discharge, upper limb paresis persisted, limiting functional use to stabilizing objects during daily activities. In contrast, lower extremity function showed marked improvement with an FMA-LE score of 19, enabling independent walking without assistive devices.

Spatial cognition assessment

Initial behavioral inattention test (BIT) score was 139 (conventional subtests: line crossing 36/36, letter cancellation 37/40, star cancellation 54/54, figure copying 3/4, line bisection 7/9, representational drawing 2/3), indicating mild USN symptoms. BIT performance showed improvement over the observation period, with total scores increasing to 146 by day 166, particularly in tasks requiring more complex spatial processing such as line bisection (from seven to nine) and figure copying (from three to four) (Table 1).

**Table 1:** Serial assessment of spatial attention from acute to recovery phase Serial assessment of spatial attention from day 32 to day 194 post-onset, including the BIT, visual attention RT using the @ATTENTION tool, and CBS. BIT total scores and subscores are shown, with higher scores indicating better performance (maximum scores: line crossing 36, letter cancellation 40, star cancellation 54, figure and shape copying four, line bisection nine, and representational drawing three). RT task results show mean RT in seconds and L/R ratio, where values closer to 1.0 indicate more balanced spatial attention. CBS scores from both observer and patient self-assessments demonstrate the discrepancy between objective and subjective evaluations of neglect symptoms (lower scores indicate fewer symptoms). The patient was assessed on days 32, 64, 101, 122, 166, and 194 after onset, corresponding to months one, two, three, four, five, and six of disease duration, respectively. BIT, behavioural inattention test; CBS, Catherine Bergego Scale; RT, reaction time; L/R ratio, left-to-right ratio

Assessment items	Day 32			Day 64			Day 101			Day 122			Day 166			Day 194		
BIT total score	139			136			140			133			146			145		
Line crossing	36			36			36			36			36			36		
Letter cancellation	37			32			35			29			40			39		
Star cancellation	54			54			54			53			54			54		
Figure and shape copying	3			3			3			3			4			4		
Line bisection	7			8			9			9			9			9		
Representational drawing	2			3			3			3			3			3		
RT task																		
RT mean	2.03			1.36			1.39			1.26			1.4			1.55		
L/R ratio	1.63			1.07			1.02			1			1.22			0.87		
CBS																		
Observer score	15			7			5			5			3			2		
Patient self-assessment score	4			2			2			1			2			2		

**Figure 4:**
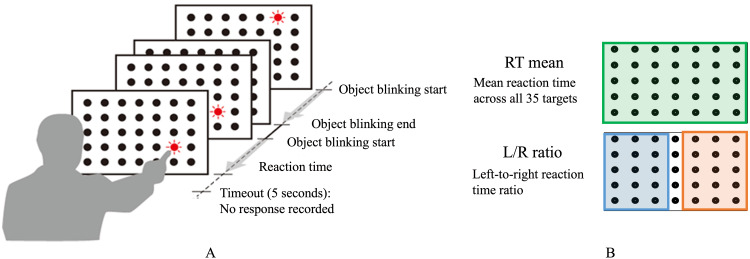
Visual attention assessment paradigm (A) Test procedure: A schematic diagram depicting the @ATTENTION tool. Each target in a 35-object grid blinks for up to five seconds, and the RT is measured from the onset of blinking until the participant responds. If no response is made within five seconds, a timeout is recorded. (B) Scoring metrics: Two primary metrics are assessed. RT mean (green box), the average RT across all 35 targets. L/R ratio (blue/orange boxes), this ratio is calculated by dividing the mean RT for 15 left-sided targets by the mean RT for 15 right-sided targets. A value greater than 1.0 indicates slower responses to left-sided targets, potentially reflecting inattention or neglect toward the left side. Note: In clinical contexts, a higher L/R ratio often suggests a relative difficulty in detecting or responding to stimuli presented on the left side of the visual field. RT, reaction time

The patient performed a custom-developed RT task using the @ATTENTION tool (Creact Corp., Tokyo) on a personal computer with a 21.5-inch touch panel display. The @ATTENTION tool (Figure 4) initially indicated delayed responses on the left side, with a left-to-right response time ratio of 1.63 and a mean RT of 2.03 seconds. This asymmetry in response times showed marked improvement during rehabilitation, with the left-to-right ratio normalizing to 1.0 by day 122 and mean RTs improving from 2.03 to 1.26 seconds, indicating recovery of spatial attention (Table 1).

The Catherine Bergego Scale (CBS) assessment showed substantial improvement in both objective and self-awareness measures. The initial assessment revealed a significant discrepancy between objective (15 points) and subjective (4 points) evaluations, indicating impaired spatial self-awareness. This discrepancy resolved over the observation period, with both objective and subjective scores improving markedly to two points each by day 194 (Table 1). The resolution of USN was evident through the improvement in BIT scores, normalization of @ATTENTION values, and reduction in CBS scores, all indicating successful recovery from spatial neglect. However, as detailed in the following section, this improvement in USN was accompanied by the emergence and persistence of hyperschematia with unique characteristics.

Hyperschematia

**Figure 5:**
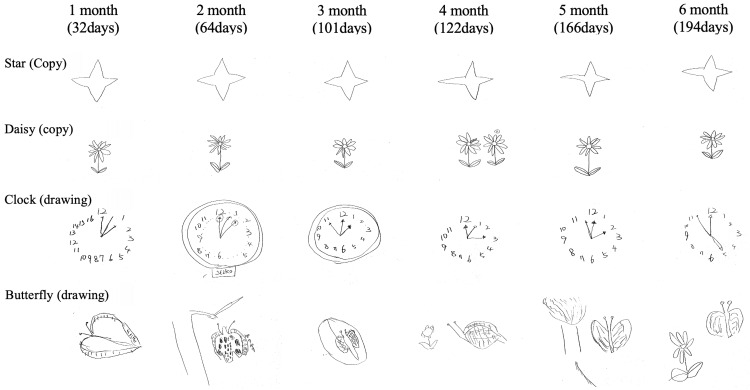
Sequential changes in copying and drawing tasks demonstrating hyperschematia Serial assessment of copying and drawing tasks from one to six months post-onset, illustrating the evolution of hyperschematia with the "over-generation" phenomenon. The tasks included copying (star and daisy) and free drawing (clock and butterfly). In the star copying task, left-sided enlargement became prominent after four months (122 days). The daisy copying task showed consistent over-generation of petals (10 instead of the model's nine) from month one to four, with an additional flower appearing on the left side at four months. The clock drawing task demonstrated significant left-sided over-generation at one month (32 days), with numbers extending to 16. The butterfly drawing task exhibited progressive addition of unrelated elements on the left side: a tree at two months (64 days), leaves at three months, a flower at four months, a tree at five months, and a flower at six months. These patterns illustrate the persistence and evolution of hyperschematia characterized by both spatial enlargement and element over-generation, particularly affecting the left side of drawings.

A distinctive feature during USN recovery was the emergence of hyperschematia in BIT tasks. Left-sided enlargement and excessive drawing became increasingly prominent, particularly at three months post-onset. The patient demonstrated notable left-sided object enlargement in the star copying task, an increased number of flower petals in copying tasks, and excessive number writing in clock drawing tasks, sometimes extending to as many as 16 numbers (Figure 5). While the patient acknowledged these drawing abnormalities when pointed out, he was initially unaware, stating, "I felt something was odd but didn't notice until it was pointed out." In subsequent assessments, he showed increased awareness, commenting, "I'll be more careful since this has been pointed out before," with notable increases in self-monitoring during testing.

Body schema and awareness assessment

Assessments for body schema disorders, including the Fluff Test (0/24), Bisach Score (0/3), and Feinberg Test (0/10), all scored zero, indicating no clear evidence of asomatognosia. However, the visual-analog test for anosognosia for motor impairment (VATAm) revealed mild anosognosia, particularly for upper limb function (initial score: 32/34). This discrepancy between subjective and objective assessments increased over time, with final scores showing a significant disparity (13/20) at three months (Table 2).

**Table 2:** Serial assessment of body schema and anosognosia Temporal changes in body schema disorders and anosognosia from day 32 to 194 post-onset. Body schema was assessed using the Bisach test, Feinberg test, and Fluff test, with scores of zero indicating no body schema disorders. The VATAm evaluated both total motor awareness and specific awareness for arm and leg impairments. VATAm scores show both observer ratings and patient self-assessments, with lower scores indicating better awareness of motor deficits. Note the consistent discrepancy between observer scores and patient self-assessment scores, particularly for arm function, suggesting mild anosognosia despite normal body schema. The hyphen (-) indicates no assessment was performed at that time point. The patient was assessed on days 32, 64, 101, 122, 166, and 194 after onset, corresponding to months one, two, three, four, five, and six of disease duration, respectively. VATAm: visual-analogue test assessing anosognosia for motor impairment

Assessment items	Day 32			Day 64			Day 101			Day 122			Day 166			Day 194		
Bisach test	0			0			0			0			0			0		
Feinberg test	0			0			0			0			0			0		
Fluff test	0			0			0			0			0			0		
VATAm																		
Observer total score	34			-			31			-			20			-		
Patient self-assessment total score	32			-			24			-			13			-		
Observer arm score	22			-			21			-			14			-		
Patient self-assessment arm score	20			-			16			-			9			-		
Observer leg score	12			-			10			-			6			-		
Patient self-assessment leg score	12			-			8			-			4			-		

Cognitive function assessment

Initial cognitive assessment revealed minimal impairment on the Mini-Mental State Examination (MMSE) with a score of 29/30 (orientation-1). The Trail Making Test (TMT) showed distinct patterns between parts A and B: TMT-A performance was consistently good throughout the assessment period with stable completion times (31-42 seconds) and no errors, while TMT-B initially showed significant impairment (109 seconds with four errors at day 32) but gradually improved (73 seconds with no errors by day 194), suggesting enhancement in divided attention and cognitive flexibility. The Behavioural Assessment of the Dysexecutive Syndrome (BADS) indicated initial executive dysfunction (profile score: 16, age-adjusted standardized score: 88), but showed substantial improvement over time (profile score: 23, age-adjusted standardized score: 123 at day 166).

Despite these improvements in standardized measures, the patient continued to demonstrate procedural errors and poor planning in daily activities, particularly when managing multiple tasks simultaneously. Examples included sequencing errors in dressing and medication management failures. While the patient was aware of these difficulties, self-correction remained challenging, necessitating verbal guidance (Table 3).

**Table 3:** Serial assessment of cognitive function Temporal changes in cognitive function from day 32 to 194 post-onset. Global cognitive function was assessed using the MMSE (maximum score 30), showing relatively preserved function (29/30 at day 32, 28/30 at day 194). Frontal lobe function was evaluated using the FAB (maximum score 18) and the BADS. FAB scores remained stable (15-17/18), while BADS performance improved over time, with age standard score increasing from 88 to 123. Attention and processing speed were measured using the TMT. TMT-A performance was consistently good with stable completion times (31-42 seconds) and no errors. TMT-B showed initial impairment (109 seconds with four errors on day 32) but improved over time (73 seconds with no errors by day 194), suggesting enhancement in divided attention and cognitive flexibility. The hyphen (-) indicates no assessment was performed at that time point. The patient was assessed on days 32, 64, 101, 122, 166, and 194 after onset, corresponding to months one, two, three, four, five, and six of disease duration, respectively. MMSE, Mini-Mental State Examination; FAB: frontal assessment battery, TMT-A, Trail Making Test-version A; TMT-B, Trail Making Test-version B; BADS, Behavioural Assessment of the Dysexecutive Syndrome

Assessment items	Day 32			Day 64			Day 101			Day 122			Day 166			Day 194		
MMSE	29			-			-			-			-			28		
FAB	15			17			16			15			16			16		
TMT-A																		
Time (seconds)	42			38			38			38			31			34		
Number of errors	0			0			0			0			0			0		
TMT-B																		
Time (seconds)	109			153			76			56			78			73		
Number of errors	4			3			0			0			0			0		
BADS																		
Total score	16			-			20			-			23			-		
Standard score	90			-			109			-			124			-		
Age standard score	88			-			108			-			123			-		

Activities of daily living assessment

**Figure 6:**
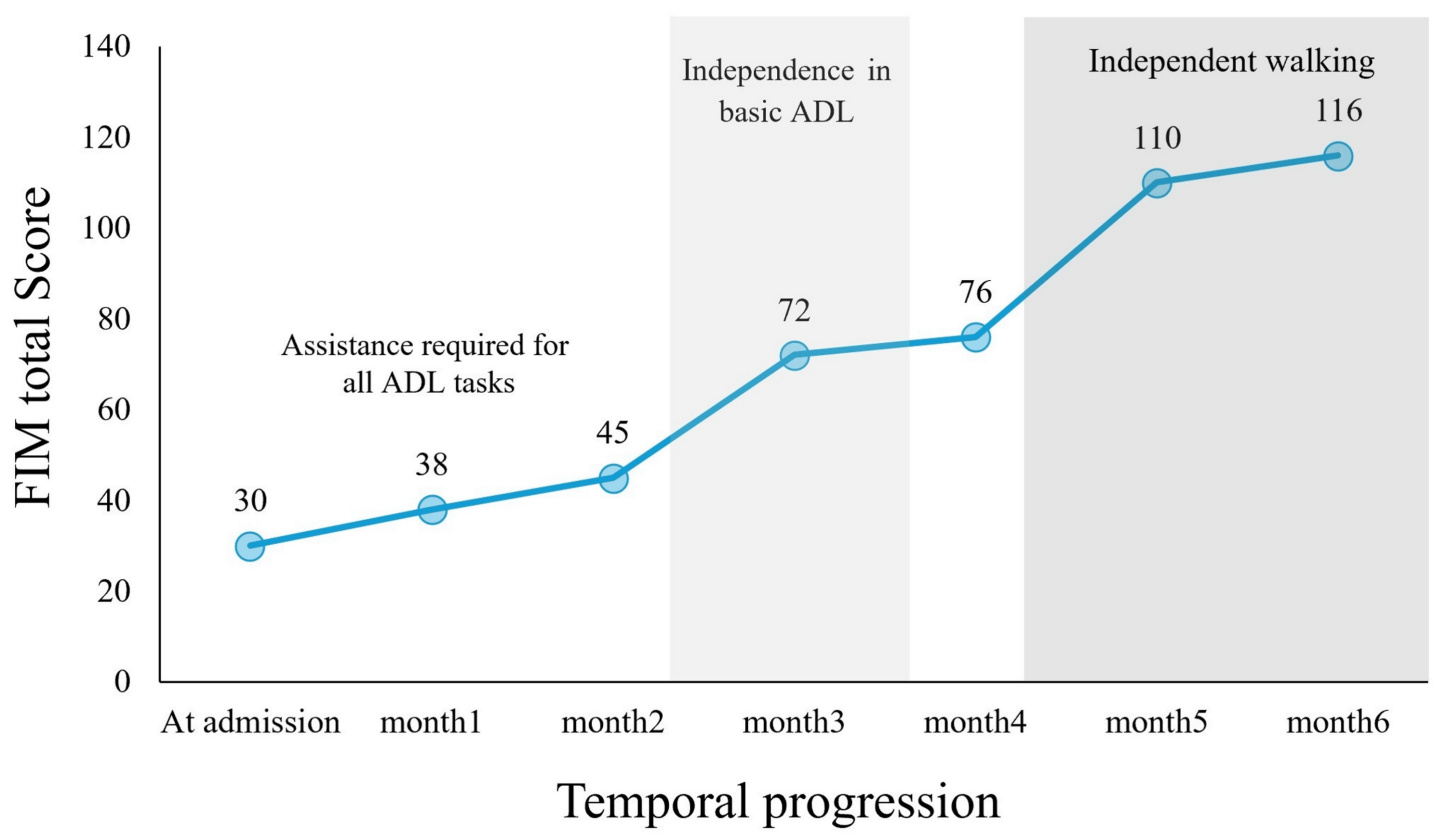
Temporal changes in FIM scores Changes in total FIM scores from admission to six months post-onset, showing three distinct recovery phases (highlighted in white and gray bands). The initial phase shows gradual improvement from admission (30 points) to month two (45 points), during which assistance was required for all ADL tasks. The second phase demonstrates the achievement of independence in basic ADLs, with scores increasing to 72 points by month three. The final phase shows rapid improvement in mobility, with the achievement of independent walking, reaching 116 points by month six. The graph illustrates a stepwise recovery pattern, with marked improvements particularly after month four. ADL, activities of daily living; FIM, functional independence measure

The functional independence measure (FIM) showed a stepwise improvement pattern across three distinct phases (Figure 6). In the initial phase, scores gradually improved from 30 points (motor: 18, cognitive: 12) at admission to 45 points at month two, during which time assistance was required for all ADL tasks. The second phase was marked by the achievement of independence in basic ADL, with scores increasing to 72 points by month three. The final phase demonstrated rapid improvement in mobility, particularly after month four, culminating in independent walking and a total FIM score of 116 points (motor: 82, cognitive: 34) by month six. Following 24 weeks of hospitalization, the patient was discharged home and immediately began a graduated return-to-work program, with full work resumption planned for 12 months post-onset.

## Discussion

This case highlights a rare scenario in which hyperschematia, characterized by over-expansion of space and "over-generation" of drawn elements, persisted even after traditional signs of USN had resolved. As illustrated in Figure 5, the patient’s overschematization became evident through enlarged and excessively detailed representations in his drawings, despite the normalization of conventional neglect measures. Conventionally, hyperschematia has been considered a compensatory or parallel phenomenon to neglect, tending to improve alongside the restoration of spatial attention [6-8]. Nonetheless, our findings contrast with this view: despite marked improvement in neglect symptoms, the patient continued to exhibit excessive spatial representation, suggesting that hyperschematia can persist independently of recovery in the dorsal stream.

From a mechanistic standpoint, Rode proposed that hyperschematia reflects a primary distortion of internal spatial mapping rather than mere inattention [9]. Vuilleumier noted that persistent positive symptoms (including hyperschematia) are seldom observed once neglect improves, underscoring the rarity of this case [10]. Our structural disconnection analysis indicates that damage to the ventral pathways, particularly the IFOF and MdLF, may have played a crucial role [11-13]. While early research emphasized dorsal-stream involvement in neglect, the IFOF and MdLF link perceptual and executive regions, and their disruption may compromise the monitoring and regulation of spatial features or object boundaries [14-18].

Clinically, the distinction between “dorsal” and “ventral” deficits is critical. The patient’s dorsal-stream deficits (manifesting as contralesional inattention) improved, as evidenced by standardized testing and daily functioning, yet the ventral pathway dysfunction persisted. This dissociation aligns with accounts suggesting that body schema and spatial representation abnormalities can arise from ventral-stream disconnections, even without overt asomatognosia in this case [19,20]. Importantly, our patient was eventually able to recognize his drawing errors, indicating partial executive awareness, but remained unable to prevent the automatic over-generation of details patterns suggesting impaired real-time feedback loops between frontal monitoring systems and ventral visual processing.

Therapeutic interventions aimed exclusively at improving spatial attention (e.g., scanning or cueing) may be insufficient for patients who exhibit persistent hyperschematia. Instead, targeted protocols emphasizing “feature monitoring” (i.e., systematic tracking of key visual elements) and “boundary maintenance” (i.e., maintaining accurate delineations of object edges and spatial limits) may be warranted. Such interventions could include systematic drawing tasks under guided feedback, explicit element counting, or boundary-setting exercises to reinforce the interplay between executive control and ventral object-processing streams. Although this is a single-case report, it illustrates the need to broaden our perspective of neglect syndromes beyond dorsal-stream deficits and to recognize that hyperschematia can endure in the absence of conventional neglect.

In conclusion, this case demonstrates that hyperschematia may persist as a distinct spatial representation disorder, uncoupled from the recovery of USN. Our findings underscore how severe ventral pathway disconnection (involving the IFOF and MdLF) can contribute to an ongoing “over-generation” phenomenon, even after neglect has largely resolved. Future research with larger samples and detailed tract-specific analyses could clarify whether similar ventral lesions consistently underlie persistent hyperschematia. Recognizing such dissociations is crucial for developing more nuanced rehabilitation strategies that address both attentional and representational components of spatial cognition.

## Conclusions

This case illustrates a rare form of persistent hyperschematia characterized by "over-generation" following right putaminal hemorrhage. Despite the resolution of USN, significant disruption of the IFOF and MdLF suggests that ventral stream disconnection may contribute to the persistence of overschematization as a distinct disorder of feature processing, rather than simply a compensatory mechanism for neglect. Specifically, the dissociation between basic object recognition (preserved) and quantity control (impaired) underscores how ventral pathways mediate real-time monitoring and regulation of spatial representation.

Clinically, these findings highlight the need for interventions beyond standard USN approaches, with an explicit emphasis on feature monitoring and boundary maintenance. Further investigation of similar cases may inform more targeted rehabilitation strategies and elucidate the neural underpinnings of hyperschematia.
